# Editorial: Recent advances in secondary diabetes and glucose intolerance

**DOI:** 10.3389/fendo.2025.1715233

**Published:** 2025-10-14

**Authors:** Ichiro Abe, Hidenori Fukuoka, Motoyuki Igata, Sujani Kodagoda Gamage

**Affiliations:** ^1^ Department of Endocrinology and Diabetes Mellitus, Fukuoka University Chikushi Hospital, Chikushino, Fukuoka, Japan; ^2^ Division of Diabetes and Endocrinology, Department of Internal Medicine, Kobe University Hospital, Kobe, Hyogo, Japan; ^3^ Department of Diabetes, Metabolism and Endocrinology, Kumamoto University Hospital, Kumamoto, Japan; ^4^ School of Medicine & Dentistry, Griffith University, Sunshine Coast, QLD, Australia

**Keywords:** secondary diabetes, diabetes mellitus, glucose intolerance, endocrinological disorder, metabolic disorder

This Research Topic encompasses current perspectives on secondary diabetes and glucose intolerance. Diabetes mellitus is characterized by hyperglycemic symptoms resulting from glucose intolerance, which is caused by impaired insulin secretion and/or elevated insulin resistance. Accurate treatment for patients with diabetes mellitus is extremely important for preventing both acute and chronic diabetic complications. While type 2 and type 1 diabetes mellitus are the most common, there is increasing recognition that treatment-refractory diabetes cases could be caused by other endocrinological or metabolic disorders, indicating secondary diabetes. Secondary diabetes is a form of diabetes mellitus that develops as a consequence of different underlying medical conditions. On the other hand, effective treatment for secondary diabetes is crucial to prevent both acute and chronic complications. Addressing the primary endocrinological or metabolic disorders can significantly improve glucose intolerance and overall diabetes management.

Commonly, a large part of secondary diabetes is caused by endocrinological disorders, such as Cushing’s syndrome (including mild autonomous cortisol secretion (MACS)), pheochromocytoma/paraganglioma (PPGL), primary aldosteronism, acromegaly, adult growth hormone deficiency, hyperthyroidism, and hypothyroidism. Recently, the mechanism of each disorder has been revealed. For instance, glucose intolerance in Cushing’s syndrome is caused by impaired insulin secretion through the glucocorticoid receptor of pancreatic beta cells as well as increased insulin resistance (1–3). Glucose intolerance in PPGL is caused by both impaired insulin secretion due to increased epinephrine secretion and increased insulin resistance due to increased norepinephrine secretion (4, 5). Hence, updated knowledge of secondary diabetes is necessary.

In this Research Topic, Costa et al. revealed that patients with non-functioning adrenal incidentalomas (NFAI), as well as those with mild autonomous cortisol secretion (MACS), have a higher incidence of glucose intolerance than those without adrenal tumors, using the oral glucose tolerance test. Previously, the report that investigated the features and diagnosis of adrenal incidentaloma showed that there were no significant differences in glucose intolerance between NFAI and MACS (6). Besides, in the previous article with meta-analysis, the patients with NFAI had a twofold higher risk of having DM than controls (7). The mechanism of glucose intolerance in NFAI remains controversial. However, the number of clinical reports, including the article in this Research Topic, revealed that patients with NFAI tend to have glucose intolerance. Thus, future investigations that clearly demonstrate the mechanism should be needed.

Regarding endocrinological secondary diabetes, Kubo et al. also reported a case with hyperglycemia due to genotype-negative multiple endocrine neoplasia type 1 (MEN1). This patient had prolactinoma, hyperparathyroidism, and MACS. Prolactinoma was treated with cabergoline, and both hyperparathyroidism and MACS were treated by surgical extirpation. Interestingly, the clinical course showed that treatment of prolactinoma with cabergoline affected the improvement of hyperglycemia. In literature, cabergoline improved hyperglycemia in patients with prolactinoma *via* improvement of insulin sensitivity (8). Hence, this report indicated that glucose intolerance with multiple endocrine disorders should be accurately evaluated and treated, leading to improved secondary diabetes.

Secondary diabetes due to metabolic disorders should be important. Nonalcoholic fatty liver disease (NAFLD) is well-known to be associated with glucose intolerance *via* increased insulin resistance (9). Liu et al. demonstrated the investigation of the predictive value of traditional and nontraditional lipid parameters in identifying abnormal glucose metabolism in NAFLD patients. The results of this investigation clarified that the levels of triglycerides (TG) and high-density lipoprotein cholesterol (HDL-C) could be associated with glucose intolerance in traditional lipid parameters. The levels of atherogenic index of plasma (AIP) and residual cholesterol (RC) could also be associated with glucose intolerance in nontraditional lipid parameters. Moreover, nontraditional lipid parameters could be superior predictive markers in identifying hyperglycemia in NAFLD patients than traditional lipid parameters. This perspective could be a standard way of predicting glucose intolerance in patients with NAFLD.

In addition, in this Research Topic, Yao et al. focused on hypoglycemia following gastrointestinal tumor surgery in patients with type 2 diabetes mellitus. It has been reported that gastrectomy with bypass reconstruction, such as Roux-en-Y gastrojejunostomy, in patients with gastric cancer could improve glucose intolerance *via* changes in the gastrointestinal hormones (10). Results of the investigation indicated five parameters (including duration of diabetes, operation duration, preoperative fasting time, preoperative hypoglycemic regimen, and glucose fluctuation on the day of surgery) could be useful for the prediction of hypoglycemia. These perspectives could contribute to gastric surgery in patients with type 2 diabetes mellitus.

In conclusion, the information presented in this Research Topic provides updated knowledge of secondary diabetes and glucose intolerance. These enrich the perspectives of secondary diabetes and glucose intolerance, which could lead to accurate treatment of treatment-refractory diabetes.
